# Putative salivary protein biomarkers for the diagnosis of oral lichen planus: a case-control study

**DOI:** 10.1186/s12903-018-0504-8

**Published:** 2018-03-13

**Authors:** Sineepat Talungchit, Waranun Buajeeb, Chotima Lerdtripop, Rudee Surarit, Kongthawat Chairatvit, Sittiruk Roytrakul, Hiroaki Kobayashi, Yuichi Izumi, Siribang-on Piboonniyom Khovidhunkit

**Affiliations:** 1 Department of Oral Biology, Faculty of Dentistry, Mahidol University, Bangkok, Thailand; 2 Department of Oral Surgery and Oral Medicine, Faculty of Dentistry, Srinakharinwirot University, Bangkok, Thailand; 3 Department of Oral Medicine and Periodontology, Faculty of Dentistry, Mahidol University, Bangkok, Thailand; 4 Department of Advanced General Dentistry, Faculty of Dentistry, Mahidol University, 6 Yodhi St., Rajthewee, Bangkok, 10400 Thailand; 5 National Center for Genetic Engineering and Biotechnology, National Science and Technology Development Agency, Pathumthani, Thailand; 6 Department of Periodontology, Graduate School of Medical and Dental Sciences, Tokyo Medical and Dental University, Tokyo, Japan; 7 Global Center of Excellence Program for Tooth and Bone Research, Tokyo Medical and Dental University, Tokyo, Japan

**Keywords:** Biomarkers, Oral lichen planus, Salivary proteins

## Abstract

**Background:**

Salivary protein biomarkers for screening and diagnosis of oral lichen planus (OLP) are not well-defined. The objective of this study was to identify putative protein biomarkers for OLP using proteomic approaches.

**Methods:**

Pooled unstimulated whole saliva was collected from five OLP patients and five healthy control participants. Saliva samples were then subjected to two-dimensional gel electrophoresis, followed by mass spectrometry to identify putative protein biomarkers. Subsequently, a subset of these putative biomarkers were validated in 24 OLP patients and 24 age-matched healthy control subjects, using an enzyme-linked immunosorbent assay (ELISA). Immunoblotting analyses were then performed in 3 pairs of age- and sex-matched OLP patients and healthy controls to confirm results from the ELISA study.

**Results:**

Thirty-one protein spots were identified, corresponding to 20 unique proteins. Notably, fibrinogen fragment D and complement component C3c exhibited increased expression in OLP patients, while cystatin SA exhibited decreased expression in OLP patients, compared with healthy control subjects. ELISA analyses indicated increased expression of fibrinogen fragment D and complement component C3c, and decreased expression of cystatin SA, in the saliva of OLP patients. Statistical differences in the expression of salivary complement C3c were observed between OLP patients and healthy control subjects. Immunoblotting analyses confirmed the results of our ELISA study.

**Conclusion:**

Complement C3c, fibrinogen fragment D and cystatin SA may serve as salivary biomarkers for screening and/or diagnosis of OLP.

## Background

Oral lichen planus (OLP) is a chronic immune-mediated disease that affects the oral mucosa. The prevalence rate of OLP is reported as 0.5–2.2% [1, 2]. A recent systematic review indicated that the malignant transformation rate of OLP-affected tissue is 1.09% [3]. There are several types of OLP, the most common of which are the reticular, atrophic, and ulcerative/erosive forms. Often, the clinical appearance of OLP mimics other types of vesiculobullous diseases; therefore, biopsy of the lesion is required for definitive diagnosis [4]. Histopathological features of OLP include hyperkeratosis, acanthosis, liquefactive degeneration of the basal cell layer, and prominent lymphocyte infiltration at the epithelium-connective tissue interface [1]. Use of direct immunofluorescent (DIF) staining also aids in the diagnosis of OLP. Recently, it was revealed that the most common manifestations found on DIF analysis of OLP biopsies are 1) shaggy expression of fibrinogen in the basement membrane zone; 2) deposition of Immunoglobulin M as colloid bodies; 3) deposition of C3 in granular and linear patterns. Thus, the presence of these proteins on DIF analysis is utilized for diagnosis of OLP [5].

Human saliva is a readily available fluid that contains locally- and systemically-produced compounds and proteins that may serve as markers for diagnosis of several diseases. Following the development of highly sensitive assays with simple and non-invasive saliva collection techniques, saliva-based biomarkers have found increasing usage in the diagnosis of disease [6]. Al-Tarawnej and colleagues performed a systematic review to define and summarize disease-related salivary biomarkers that could be identified by mass spectrometry proteomics [7]. These diseases included Sjögren’s syndrome, squamous cell carcinoma, diabetes, breast cancer, dental caries, periodontitis, systemic sclerosis, bleeding oral cavity, graft versus host disease and OLP. A total of 180 biomarkers were identified; 87 of these were upregulated, 63 were downregulated, and 30 varied in a disease-dependent manner [7].

Some proteins have been previously reported as salivary biomarkers for OLP. Notably, C-reactive protein, Immunoglobulin A (IgA), matrix metalloproteinase 8, CTX I, CD14 and toll-like receptor-2 demonstrate upregulation in the saliva of OLP patients [8–11]. Thus far, very few studies have used a proteomic approach to identify potential salivary protein biomarkers for OLP [12–14]. In one of these, Mizukawa et al. used reverse-phase high-performance liquid chromatography and mass spectrometry (MS); they reported a significant increase in defensin-1 (HNP-1) levels in the saliva of patients with OLP, compared with HNP-1 levels in healthy subjects [12]. In another study, Yang et al. evaluated potential biomarkers in whole saliva from OLP patients [13]. They identified 14 proteins with at least a two-fold difference in intensity between OLP patients and healthy controls. Importantly, urinary prokallikrein was elevated, while palate, lung, and nasal epithelium carcinoma-associated protein (PLUNC) was reduced in saliva samples from OLP patients. Lastly, Chaiyarit et al. used matrix assisted laser desorption/ionization time-of-flight/time-of-flight (MALDI-TOF/TOF) MS to analyze the patterns of mass signals from low-molecular-weight proteins that were present in saliva samples from patients with oral cancer, OLP, and chronic periodontitis [14]. Mass signals at both 12,964.55 and 13,279.08 Da were significantly elevated in the saliva of OLP patients, compared with the other groups. However, the corresponding proteins have not been identified.

Though numerous candidate proteins have been discovered, these must be validated in other patient populations. The identification of potential salivary protein biomarkers is an essential first step in the development of effective screening techniques and early diagnosis of OLP, which is expected to improve treatment outcomes and quality of life for affected patients. The objective of this study was to use proteomics approaches to identify potential salivary protein biomarkers for screening and diagnosis of OLP.

## Methods

### Study subjects

Human saliva was collected from patients at the Faculty of Dentistry of Mahidol University, under guidelines set by the Faculty of Dentistry/Faculty of Pharmacy, Mahidol University, Institutional Review Board (MU-DT/PY-IRB 2011/012.3103) for the Use of Human Subjects in Research. A written informed consent was acquired from each individual.

All patients were diagnosed with OLP using the modified World Health Organization diagnostic criteria for OLP, as set forth by van de Meij and van der Waal [15]. All patients exhibited symmetrical distribution of the lesions on the bilateral buccal mucosa, gingiva or both lateral borders of the tongue. Histopathology also confirmed diagnosis of OLP; all included specimens presented with a band-like zone of cellular infiltration—primarily composed of lymphocytes—liquefaction degeneration in the basal cell layer, and an absence of epithelial dysplasia. Some patients reported a history of medication use, but the medication was either not known to induce oral lesions or was started after the eruption of oral lesions.

Two separate sets of patients were used in this study, to allow extrapolation of our results from the first set of patients to the second set of patients. The second set of patients exhibited similar clinical and pathological characteristics of OLP, thus demonstrating that these proteins could be used as markers for OLP. The first set included five patients with OLP and five sex-matched healthy control participants. Saliva samples from the five OLP patients and five healthy control participants in the first set were separately pooled, then subjected to two-dimensional polyacrylamide gel electrophoresis (2D-PAGE) followed by MS. The second set of patients was recruited for the validation of the results from the first set of patients; for the second set of patients, we used enzyme-linked immunosorbent assay (ELISA) and immunoblotting analyses. This second set of participants was composed of 24 OLP patients and 24 age-matched control subjects.

We included participants in the age range of 30–75 years old. Healthy control subjects comprised individuals who exhibited normal mucosa and stated that they had no underlying systemic diseases. Exclusion criteria included positive laboratory detection of *Candida* spp. or presence of periodontitis; we also excluded patients who currently smoked, were pregnant, had been treated with radiation therapy, or had other diseases that might affect salivary production, such as Sjögren’s syndrome and cystic fibrosis. Clinical presentation of OLP was characterized using a Reticular-Erythematous-Ulcerative (REU) system [16].

### Saliva sample collection and preparation

Whole unstimulated saliva (WUS) was collected using standard methods [17]. Briefly, samples were collected between 9 am and 12 am. Subjects were asked to avoid eating, drinking or using oral hygiene products for ≥1 h before sample collection. They were also asked to rinse their mouths with plain water prior to collection. During the collection, subjects were asked to swallow first, tilt their head forward and expectorate all saliva into a 50-mL centrifuge tube without swallowing until the volume was 5 ml. The time needed to collect the entire sample of saliva was recorded. Saliva was immediately placed on ice and subsequently centrifuged at 2600 × g at 4 °C for 15 min to remove debris and cells. A protease inhibitor cocktail (Roche Diagnostics GmbH, Mannheim, Germany) was added to the supernatant, which was then aliquoted into smaller volumes and stored at − 80 °C until use.

### Two-dimensional gel electrophoresis-based proteomics

First, we performed 2D-PAGE on five pairs of individual saliva samples from OLP patients and healthy control participants. Although we observed similar trends of differentially expressed proteins, we found some non-redundant protein spots that were differentially expressed in these gels. To ensure that all non-redundant protein spots underwent further analysis, we performed 2D-PAGE in triplicate, using pooled saliva samples from these patients and controls, and selected the best pair of gels for further analysis. Therefore, an equal amount of salivary protein from five subjects of OLP and five healthy control subjects was pooled; a total of 250 μg of the pooled salivary protein was then cleaned via acetone precipitation and resuspended in a rehydration solution at a final volume of 125 μL. The 2D-PAGE was performed according to the method of Sukprasert et al., 2013 [18], with a slight modification where protein samples were loaded onto 7 cm, linear pH 3–10 IPG strips (GE Healthcare, Buckinghamshire, UK). After samples were rehydrated for 16 h isoelectric, focusing (IEF) was conducted using the Ettan IPGphor 3 System (GE Healthcare, Buckinghamshire, UK). After reduction and akylation steps were completed, the equilibrated gel strips were applied to an upright 12% gel polyacrylamide sodium dodecyl sulfate (SDS-PAGE) and electrophoresis was conducted for the second dimension. The gels were stained using Coomassie Brilliant Blue G250, then scanned and analyzed with ImageMaster™ 2D Platinum software version 7 (GE Healthcare, Buckinghamshire, UK).

The intensity of each protein spot was measured by integration of the background-subtracted pixel intensity in a spot area distant from the selection. Images were normalized and analyzed; differentially expressed proteins were identified and quantified. Only spots with ≥1.5-fold difference in abundance, or spots that were found exclusively in either group were recorded as differentially expressed proteins that were then chosen for further MS analysis.

### In-gel digestion and liquid chromatography coupled with tandem mass spectrometry (LC-MS/MS) and protein identification

These procedures were performed as previously described [19, 20]. Briefly, protein spots were excised, reduced, alkylated and trypsinized overnight at 37°C. After extraction and washing, nanoscale LC separation of tryptic peptides was performed using an Ultimate 3000 LC System (Dionex, USA) that was coupled to an ESI-Ion Trap MS (HCTultra PTM Discovery System, Bruker, Germany). The analyzed MS/MS data were submitted to a database search that used a local Mascot server with specific parameters. The Mascot results were used as queries for protein identification searches in the National Center for Biotechnology Information (NCBI) database. Proteins that met our criteria for “identified proteins” exhibited ≥1 peptide with an individual Mascot score of *p* < 0.05.

### Enzyme-linked immunosorbent assay (ELISA)

WUS from an independent set of samples from patients with OLP and healthy control subjects were collected and prepared as described above. The amino acid sequence of chain B, fibrinogen fragment D, detected from the 2D-PAGE and LC-MS/MS belongs to the fibrinogen β chain; therefore, an ELISA kit that detected fibrinogen β chain was used in this study. Salivary complement component C3c, fibrinogen fragment D, and cystatin SA levels were evaluated in the saliva samples using commercially available ELISA kits for human C3c (Hycult Biotech, PB Uden, The Netherlands), human fibrinogen β chain, and cystatin SA (LifeSpan BioSciences, Seattle, WA, USA) in accordance with the manufacturers’ instructions. A microplate reader (VMax Kinetic ELISA Microplate Reader, Molecular Devices Corporation, Sunnyvale, CA, USA) was used to measure absorbance at 450 nm. Protein contents were expressed in ng/mL, and the minimum detectable levels of each ELISA kit were quantified in accordance with each corresponding manufacturer.

### Immunoblotting analysis

Three pairs of individual WUS samples from the same set used in the ELISA analysis were randomly selected and used for immunoblotting analysis. The protein concentration was determined using the BCA™ protein assay kit (Pierce, Rockville, IL, USA), according to the manufacturer’s protocol. Five μg of total protein in each individual sample was separated by 8% or 12% SDS-PAGE. The separated proteins were transferred to a nitrocellulose membrane and subsequently probed with one of the following antibodies: polyclonal rabbit anti-human C3c complement antibody (DAKO, Glostrup, Denmark); polyclonal goat anti-fibrinogen β antibody (Santa Cruz Biotechnology, Inc.USA,); monoclonal mouse anti-cystatin SA antibody (Abcam, Cambridge, UK). Subsequently, the membranes were incubated with one of the following antibodies: for C3c, horseradish peroxidase-conjugated goat anti-rabbit IgG secondary antibody (Bio-Rad Laboratories, Inc.USA ,); for fibrinogen β, horseradish peroxidase-conjugated donkey anti-goat IgG secondary antibody (Bio-Rad Laboratories, Inc., USA); for cystatin SA, horseradish peroxidase-conjugated goat anti-mouse IgG secondary antibody (Santa Cruz Biotechnology, Inc., USA). Immunoreactive protein bands were visualized using Western Lightning® Plus-ECL substrate (PerkinElmer, Inc., Waltham, MA, USA).

### Statistical analysis

Descriptive statistics were used to analyze 2D-PAGE. Protein quantification and identification and ELISA were first analyzed for normal distribution using the Kolmogorov-Smirnov test; at this stage, the homogeneity of variances was also tested. If the data were normally distributed and the variance was homogeneous, Student’s *t*-test was used to analyze the differences among the study groups. If the data did not follow a normal distribution, the Kruskal-Wallis test was used to check for any significant differences among the study groups; differences among groups were then verified using the Mann-Whitney U test. Statistical significance was set at *p* < 0.05. Log transformations were performed to permit approximation of a normal distribution of the data; this was required to calculate correlations. The results are displayed as the median and interquartile range (25th and 75th percentile).

## Results

### Two-dimensional gel electrophoresis-based proteomics

The characteristics of the five OLP patients and five sex-matched healthy control subjects are presented in Table 1. The OLP patient group was significantly older (*p* = 0.007).

**Table 1:** Demographic characteristics and clinical features of oral lichen planus patients and healthy control subjects in the two-dimensional polyacrylamide gel electrophoresis/proteomics portion of the study

	OLP (*n* = 5)	Healthy control (*n* = 5)	*p*
Age (years)			
Mean ± SD	56.8 ± 4.09	47.8 ± 3.9	*0.007*
Range	52–61	44–53	
Gender			
F/M	4/1	4/1	
Clinical appearance			
R & E	3 (60%)		
R & E (with erosion)	1 (20%)		
R & E & U	1 (20%)		
Site			
Buccal mucosa	1 (20%)		
Gingiva	2 (40%)		
Buccal mucosa and gingiva	2 (40%)		

*SD* Standard Deviation, *R* Reticular/hyperkeratotic lesions, *E* Erosive lesions, *U* Ulcerative lesions, *OLP* oral lichen planus

**Fig. 1:**
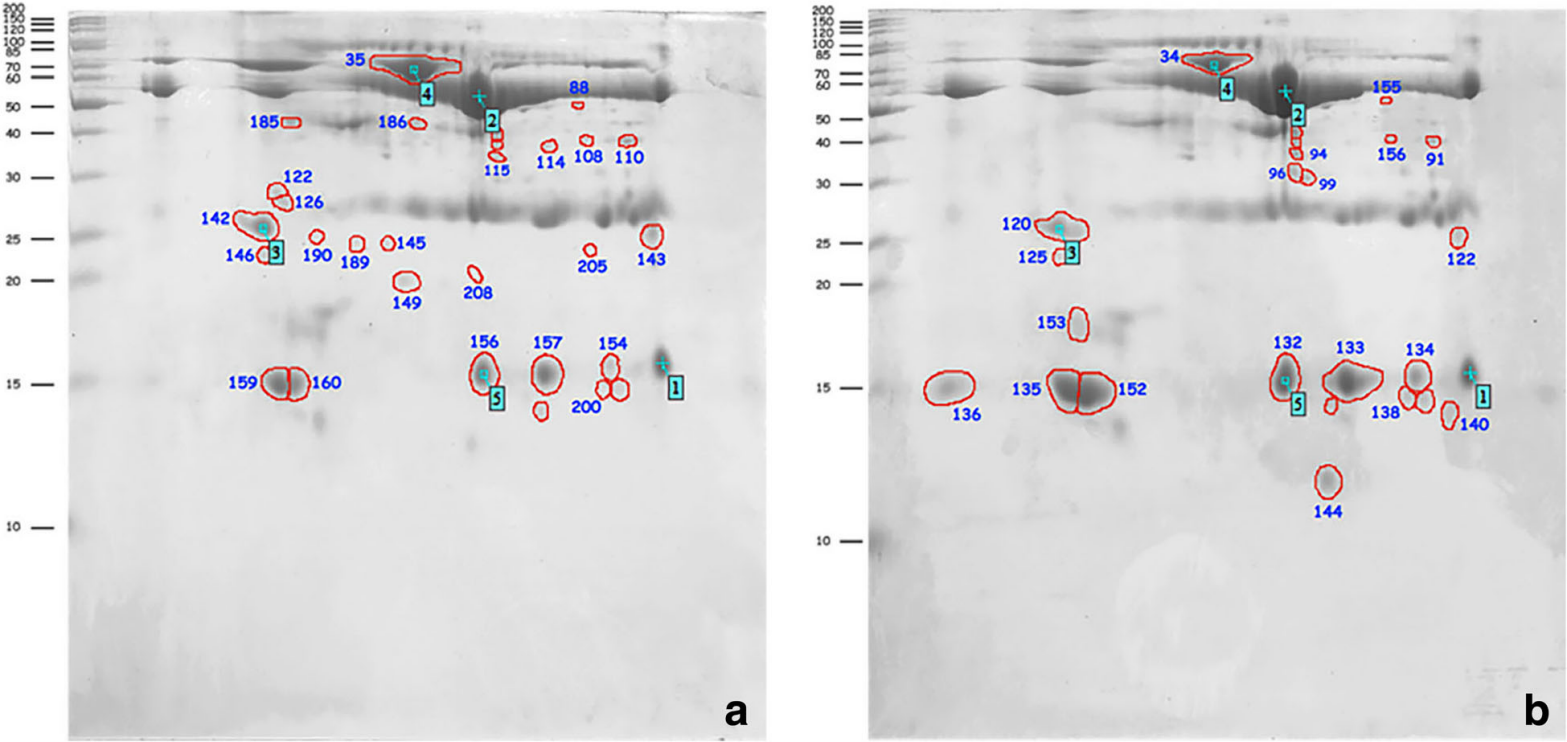
Two-dimensional polyacrylamide gel electrophoresis images of the pooled salivary proteins from the oral lichen planus (OLP) group (**a**) and pooled healthy control group (**b**). **a**, **b** The identification of 31 protein spots that either demonstrated a ≥ 1.5-fold difference in abundance or were found exclusively in one group (OLP or healthy control) is demonstrated

Saliva samples from the OLP and control groups were pooled using an equal amount of protein from each sample within each group. Representative gels from the OLP and healthy control groups are shown in Fig. 1. Over 100 protein spots were detected on Coomassie Blue-stained gels; image analysis identified 31 protein spots that exhibited ≥1.5-fold difference in abundance or were found exclusively in either the OLP or healthy control groups. Of these 31 protein spots, 14 protein spots (likely representing nine or 10 unique proteins) were found in both the OLP and healthy control groups (Table 2). Two spots (numbers 135 and 152) could not be identified as either a cystatin S precursor or cystatin SA. In addition, 11 protein spots (representing seven unique proteins) were detected only in the OLP group (Table 3), and six other protein spots (representing five unique proteins) were present only in the healthy control group (Table 4). In the 14 matched protein spots that were found in both the OLP and healthy control groups, seven protein spots (representing six unique proteins) exhibited increased expression in the OLP group, whereas seven other protein spots (likely representing three or four unique proteins) exhibited decreased expression in the OLP group.

**Table 2:** Human salivary proteins that exhibited significant differences in abundance between oral lichen planus patients and healthy control subjects

Spots ID	Protein name	Accession no.	Protein MW (kDa)	Protein pI	Protein score	Sequence coverage (%)	Fold change
34, 35	Serum albumin	gi|28,592	71.3	6.05	2085	65	3.0
88, 155	Alpha-amylase	gi|178,585	58.4	6.32	48	3	2.1
91, 110	Glyceraldehyde-3-phosphate dehydrogenase	gi|31,645	36.2	8.26	311	24	2.8
108, 156	Short-chain specific acyl-CoA dehydrogenase, mitochondrial precursor	gi|4,557,233	44.6	8.13	23	7	2.6
120, 142 125, 146	Ig J-chain, partial	gi|532,598	16.1	4.62	262 124	32 27	2.0 2.4
122, 143	Neutrophil gelatinase-associated lipocalin, NGAL	gi|300,181	20.7	8.87	344	55	3.2
94, 115	Carbonic anhydrase isozyme VI	gi|179,732	35.5	6.65	131	11	-1.8
132, 156 133, 157 134, 154 138, 200	Cystatin precursor	gi|337,752	16.6	6.82	437 451 192 151	45 45 31 43	-1.5 -2.1 -3.4 - 1.6
135, 159	Cystatin S precursor, Cystatin SA	gi|4,503,109, gi|359,513	16.5, 14.1	4.95, 4.85	377, 257	60, 57	-1.6
152, 160					420, 401	49, 57	-3.2

(-) fold change: under-expressed in OLP compared with healthy controls

**Table 3:** Human salivary proteins that were identified exclusively in oral lichen planus patients

Spots ID	Protein name	Accession no.	Protein MW (kDa)	Protein pI	Protein score	Sequence coverage (%)
114	NOL1/NOP2/Sun domain family, member 4	gi|15,680,185	43.6	8.47	31	2
122, 126	14–3-3 protein zeta/delta	gi|4,507,953	27.9	4.73	57, 150	10
145, 189, 190, 205	PREDICTED: uncharacterized protein LOC101926929	gi|530,365,914	23.1	9.2	52, 45, 49, 52	–
149	Haptoglobin	gi|3,337,390	38.7	6.14	98	9
185	Chain C, Human Complement Component C3c	gi|78,101,271	40.2	4.79	236	16
186	Chain B, Crystal Structure of Fibrinogen Fragment D	gi|2,781,208	38.1	5.84	50	5
208	Cytokeratin 9	gi|435,476	62.3	5.19	61	3

**Table 4:** Human salivary proteins that were identified exclusively in healthy control subjects

Spots ID	Protein name	Accession no.	Protein MW (kDa)	Protein pI	Protein score	Sequence coverage (%)
96, 99	Carbonic anhydrase isozyme VI	gi|179,732	35.5	6.65	186, 32	11, 4
136	Cystatin S	gi|235,948	14.4	4.74	121	27
140	Cystatin precursor	gi|337,752	16.6	6.82	89	31
144	Unnamed protein product	gi|29,888	11.0	9.19	91	23
153	Prolactin-induced protein	gi|116,642,259	9.2	5.25	199	37

Table 5 lists the functions of each protein that was differentially expressed in OLP patients, compared with healthy controls. The possible relationship of each protein to OLP is also described (Table 5). Notably, 4 of the proteins, namely, serum albumin, fibrinogen, complement C3 and alpha amylase have been investigated in studies of OLP patients. Two proteins including haptoglobin and Neutrophil-gelatinase associated lipocalin (NGAL) have been examined in skin lichen planus (LP). OLP is considered an immune-mediated disease and a potentially malignant disorder; therefore, where there was no direct association between the proteins and OLP disease, we used other related autoimmune or dysplastic oral diseases for the report.

**Table 5:** A summary of the function of each salivary protein identified by proteomics in this study, and its association with lichen planus in previous studies

Protein name	Functions	Relation of protein to OLP
Serum originating proteins		
Serum albumin	Its main function is the regulation of the colloidal osmotic pressure of blood [44]. It also serves as antioxidant agent [45].	In a study of Battino, et al., serum and salivary anti-oxidant proteins including albumin were investigated using analysis kits from Diasys (Germany). No significant differences were observed both in saliva and in serum for the albumin values in OLP patients when compared with the control group. Even if albumin is known as a protein with antioxidant properties [46]. In another study, the serum albumin levels of OLP patients were within the normal range [47].
Haptoglobin	Haptoglobin captures and combines with free plasma hemoglobin to allow hepatic recycling of heme iron [48]. It is an acute phase reactant which involves in acute phase immune response [49]. It can be detected in the cytoplasm of normal epidermal Langerhans cells and epidermal keratinocytes [50].	In situ hybridization and immuno histochemistry revealed increased haptoglobin expression in keratinocytes of the skin of patients with psoriasis, lichen planus, erythroderma and seborrheic keratosis [21].
Chain B, crystal structure of fibrinogen fragment D	Fibrinogen is a glycoprotein which plays important roles in blood clotting, fibrinolysis, cellular and matric interactions, cell adhesion, inflammatory response, wound healing process and neoplasia [51, 52].	Oral epithelial cells may synthesize and secrete fibrinogen locally in response to proinflammatory mediators [34]. Buajeeb et al. found that using immunofluorescent technic for the detection of fibrinogen in oral lichen planus lesions, 98.5% of tissue specimens were positive for fibrinogen [5]. Other studies also showed presence of fibrinogen in OLP tissue samples using direct immunofluorescence [53, 54].
Chain C, human complement component C3c	C3 plays a central role in the activation of the complement system which mediates local inflammatory process [35]. It is derived from proteolytic degradation of complement C3 and is the major stable conversion product of complement C3 [36].	In one study, component C3 levels were determined in serum and saliva of patients with OLP compared to normal controls. The C3 levels were increased in the OLP group compared with the control group (*p* < 0.05) [55]. In addition, the presence of C3 deposition as granular and linear patterns in the immunofluorescence has been utilized for the diagnosis of OLP [5].
Gland originating salivary proteins		
Alpha amylase	An enzyme that plays a role in the initial digestion of starch [56, 57].	Salivary alpha amylase levels were higher in the morning in patients with OLP compared to control subjects. However, in the evening these levels were lower in OLP subjects than control subjects [58].
Carbonic anhydrase isozyme VI	It has been suggested that carbonic anhydrase VI participates in the maintenance of appropriate pH homeostasis on tooth surfaces as well as in the mucosa of the gastrointestinal canal [59].	No report about this protein in OLP or other related diseases.
Cystatin S and SA	They are proteins belongs to the cystatin superfamily which are cysteine protease inhibitors usually expressed in saliva, tears, urine, and seminal fluid [60]. The S-type cystatins (cystatin S, SN and SA) are mostly found in saliva and share a large intensity of amino acid similarity [61]. They also exert some role in the regulation of saliva calcium and antimicrobial activity [61, 62].	Cathepsin L has been found to be increased in skin specimens of patients with LP [40]. It has been found that salivary cystatin SA can inhibit human cathepsin L [41] which may modulate proteolytic events in OLP.
Prolactin-induced protein	It is a small protein functions in human reproductive and immunological system. It is expressed in several exocrine tissues such as the lacrimal, salivary, and sweat glands [63].	No report about this protein in oral lichen planus or other related diseases.
Tissue/mucosal originating proteins		
Glyceraldehyde-3-phosphate dehydrogenase	It is an enzyme that involves in glycolysis. It has recently been reported to be implicated in transcription activation, initiation of apoptosis [64], ER to Golgi vesicle shuttling, and fast axonal, or axoplasmic transport [65].	No report about this protein in oral lichen planus or other related diseases.
14–3-3 protein zeta/delta	It binds to several proteins and is involved in signal transduction process, regulation of apoptosis and membrane organization [25]. 14–3-3 delta is the phosphorylated form of 14–3-3 zeta [26].	Immunohistochemistry confirmed overexpression of 14–3-3 zeta and theta in premalignant oral lesions and oral squamous cell carcinoma tissues in comparison with normal epithelium [27]. Since oral lichen planus is considered a premalignant lesion, there is a possibility that this protein might be increased in this disease. 14–3-3 zeta/delta has been found to be increased in rheumatoid arthritis, an autoimmune disease [66]. 14–3-3 sigma, an isoform of 14–3-3 protein is overexpressed in oral lichen planus on immunohistochemical analysis [67].
Short-chain specific acyl-CoA dehydrogenase, mitochondrial precursor	The short-chain specific acyl-CoA dehydrogenase enzyme catalyzes the first part of fatty acid beta-oxidation by forming a C2-C3 trans-double bond in the fatty acid through dehydrogenation of the flavoenzyme. It is specific to short-chain fatty acids, between C2 and C3-acyl-CoA [68]. When there are defects that result in this enzyme being misfolded, there is an increased production of reactive oxygen species (ROS); the increased ROS forces the mitochondria to undergo fission, and the mitochondrial reticulum takes on a grain-like structure [69].	No report about this protein in oral lichen planus or other related diseases.
Neutrophil gelatinase-associated lipocalin, NGAL	It is a small cationic antimicrobial peptides of epithelial origin [70]. It functions in innate defense against microbial agents [71]. The ligands of NGAL are a variety of bacteria ferric siderophores, which transport iron to bacteria [72]. By taking the iron away from bacteria, NGAL acts as a potent bacteriostatic agent under iron-limiting conditions [73].	NGAL has been found to be a marker for dysregulated keratinocyte differentiation in human skin [74]. Interestingly, NGAL expression was highly increased in psoriasis-like inflammatory disorders such as lichen planus and pityriasis rubula pilaris and skin cancers including keratoacanthoma and squamous cell carcinoma implying that NGAL may be related with the epidermal hyperplasia [22].
NOL1/NOP2/Sun domain family, member 4 (NSUN4)	It is a 5-methylcytosine RNA methyltransferase [75]. Binding of this protein to mitochondrial transcription termination factor 4 (MTERF4) is crucial for ribosome biogenesis which is critical for oxidative phosphorylation capacity. Biogenesis of mammalian mitochondrial ribosomes requires a maturation of both the small and large ribosomal subunit and NSUN4 in complex with MTERF4 is required to assemble the small and large subunits to form a monosome [76].	No report about this protein in oral lichen planus or other related diseases.
Cytokeratin 9	Cytokeratins are proteins expressed in epithelial cells. It has been used for the markers of epithelial cell differentiation and have been used for determining tumor origin and epithelial cell typing [77].	In a study of Boisnic et al., cytokeratin expression was determined by means of immunohistochemical labeling with use of monoclonal antibodies against cytokeratins and filaggrin and two-dimensional gel electrophoresis. In buccal mucosa lichen planus, the appearance of cytokeratins 1, 2, 10, and 11 coincides with a decrease in cytokeratins 4 and 13 and a moderate increase in cytokeratins 6, 16, 17, and 19. Moreover, this work showed that cytokeratins 1, 2, 10, and 11 and filaggrin are sensitive tools that may help detect early relapse of OLP before clinical exacerbation [78].
Ig J-chain, partial	The J Chain is required for IgM or IgA to be secreted into mucosa [23]. As part of a polymeric immunoglobulin, the J-chain is essential for the formation of dimeric IgA and pentameric IgM molecules and facilitates the secretory component upon excretion by epithelial cells [23]. It also modulates the activation of complement system [23].	No report directly investigate the role of IgJ in OLP. However, Lopez-Jornet has reported significantly increased levels of salivary IgA in patients with OLP compared to normal control [8]. In addition, there is a study indicated the increase level of IgJ mRNA in oral dysplastic lesions [79].

From these results, we selected three proteins for further biomarker validation according to their roles in the biological process of OLP and their overall molecular functions. These proteins were cystatin SA, chain C of human complement component C3c, and chain B of fibrinogen fragment D. All three proteins were tested by ELISA and immunoblotting analysis to validate their usage as biomarkers in other individual samples.

### Validation of potential biomarkers by ELISA

We evaluated saliva from 24 OLP patients and 24 age-matched healthy control subjects. The characteristics and clinical features of these subjects are summarized in Table 6. Salivary levels of complement component C3c, fibrinogen fragment D, and cystatin SA are also described in Table 6. Salivary levels of complement component C3c exhibited significant elevation in the OLP group, compared with the healthy control group (*p* = 0.041) (Table 6). There was weak statistical evidence that salivary levels of fibrinogen fragment D were higher, and salivary levels of cystatin SA were lower, in the OLP group, compared with the healthy control group (*p =* 0.398 and *p =* 0.281 for the respective comparisons).

**Table 6:** Demographic characteristics and clinical features of oral lichen planus patients and healthy control subjects in the validation portion of this study

	OLP (*n* = 24)	Healthy control (*n* = 24)	*p*
Age (years)			
Mean ± SE	57.25 ± 12.41	51.79 ± 11.71	*0.124*
Range	30–76	33–70	
Gender			
F/M	20/4	22/2	
Clinical appearance			
R & E	16 (66.7%)		
R & E (with erosion)	2 (8.3%)		
R & E & U	6 (25%)		
Site			
Buccal mucosa	6 (24.9%)		
Buccal mucosa and gingiva	7 (29.05%)		
Buccal mucosa and tongue	1 (4.15%)		
Buccal mucosa, labial mucosa and gingiva	1 (4.15%)		
Buccal mucosa, gingiva and tongue	2 (8.3%)		
Gingiva	4 (16.6%)		
Gingiva and palate	1 (4.15%)		
Palate	1 (4.15%)		
Palate and labial mucosa	1 (4.15%)		
Salivary C3c median levels (ng/ml) (Q1, Q3)	13.53 (7.87, 27.36)	9.91 (4.60, 16.97)	
Salivary FGD median levels (ng/ml) (Q1, Q3)	9.65 (5.47, 18.76)	9.60 (4.48, 17.77)	
Salivary cystatin SA median levels (ng/ml) (Q1, Q3)	2.95 (1.52, 3.72)	4.12 (1.38, 6.66)	
Salivary C3c log mean ± SD levels (ng/ml)	1.21 ± 0.41	0.95 ± 0.43	*0.041*
Salivary FGD log mean ± SD levels (ng/ml)	1.01 ± 0.38	0.90 ± 0.47	*0.398*
Salivary cystatin SA log mean ± SD levels (ng/ml)	0.39 ± 0.23	0.51 ± 0.46	*0.279*

*SD* Standard Deviation, *R* Reticular/hyperkeratotic lesions, *E* Erosive lesions, *U* Ulcerative lesions, *C3c* complement C3c, *FGD* fibrinogen fragment D, *OLP* oral lichen planus

### Validation of potential biomarkers by immunoblotting analysis

**Fig. 2:**
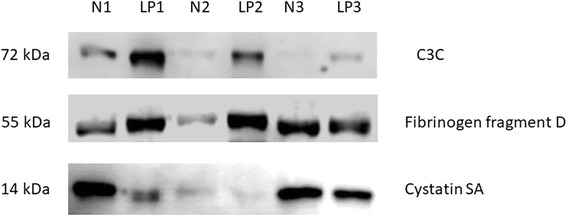
Immunoblotting analysis of salivary proteins (complement C3c, fibrinogen fragment D, and cystatin SA) was performed in 3 pairs of age- and sex-matched oral lichen planus patients (LP1/2/3) and healthy control subjects (N1/2/3)

Since there are limitations in using ELISA for detection of these proteins in saliva, we subsequently investigated protein expression by immunoblotting analysis. For this validation, we randomly selected three pairs of patients and controls from among the 24 age-matched OLP patients and healthy control subjects. Figure 2 demonstrates the results of immunoblotting analysis of the expression of these three proteins in the saliva of OLP patients, compared with healthy controls. We found increased expression of C3c and fibrinogen fragment D, and decreased expression of cystatin SA, in the saliva of OLP patients, compared with healthy control subjects. These results were consistent with our ELISA results.

## Discussion

In the present study, we used a 2D gel-based proteomic approach to determine the protein profile of WUS from OLP patients (Fig. 1). We reviewed and reported functions of the proteins that were differentially expressed between the saliva of OLP patients and healthy control subjects (Table 5). We then selected three proteins that appear to have strong potential to serve as biomarkers for OLP and validated the salivary levels of these proteins through ELISA and immunoblotting analyses (Tables 2, 3, 4 and Fig. 2). We found that fibrinogen fragment D and complement component C3c exhibited increased expression in the saliva of OLP patients (Table 6 and Fig. 2). Moreover, we found that cystatin SA exhibited decreased expression in the saliva of OLP patients, compared with healthy control subjects (Table 6 and Fig. 2).

Using a 2D gel-based proteomic approach, we found 31 protein spots with either a ≥ 1.5-fold difference in abundance or an exclusive presence in one of the study groups (OLP patients or healthy controls); these spots were excised for further identification by LC-MS/MS. We ultimately identified 20 unique proteins, of which 13 exhibited increased expression in OLP patients, compared with healthy control subjects (Tables 2 and 3). In contrast, the remaining seven proteins exhibited decreased expression in OLP patients (Tables 2 and 4).

Some of the proteins that were identified in this study have been previously reported to exhibit increased expression in patients with oral or skin LP. For example, C3 and fibrinogen have been used for the diagnosis of OLP on immunofluorescent analysis [5]. Haptoglobin, an acute phase reactant protein, and NGAL, a small cationic antimicrobial peptide of epithelial origin and marker for dysregulated keratinocyte differentiation, both demonstrated increased expression in keratinocytes from the skin of patients with LP [21, 22]. The Immunoglobulin J (IgJ) chain is required for dimerization of IgA molecules, allowing them to be secreted as secretory-IgA (sIgA) [23]. Several studies have described increased sIgA expression in the saliva of OLP patients; this is consistent with our detection of increased IgJ expression in the saliva of our cohort of OLP patients [8, 24].

Another protein upregulated in OLP patients, 14–3-3 zeta/delta, has multiple roles in signal transduction, regulation of apoptosis, and oral carcinogenesis [25, 26]. Immunohistochemical analysis has demonstrated overexpression of this isoform of 14–3-3 in premalignant oral lesions and samples of oral squamous cell carcinoma [27, 28]. Using proteomics integrated with bioinformatics to compare metastatic and non-metastatic cancer cell lines, multiple interaction partners of 14–3-3 zeta have been identified, suggesting its involvement in several intracellular protein networks, including evasion of apoptosis, cytoskeletal remodeling, protein synthesis, protein folding/degradation, and nuclear import and export of proteins in oral cancer cells [29]. Since OLP is a potentially malignant disorder, the presence of this protein in the saliva of OLP patients may contribute insights on how OLP may transform into oral cancer.

Several protein biomarkers have been previously reported for the diagnosis of OLP by proteomic analysis. These include salivary CD14, urinary prokallikrein, PLUNC, and defensin-1 [9, 12–14]. In addition, two unidentified mass signals, with molecular weight values of approximately 12.9 and 13.2 kDa, are significantly increased in the saliva of OLP patients [14]. Importantly, different techniques for the preparation of salivary samples are likely to influence the detection of various proteins in the saliva. Yang et al. detected an increase in urinary prokallikrein levels, and a decrease in PLUNC levels in saliva samples from OLP patients, using 2-DE followed by MALDI-TOF MS [13]; however, these two proteins were not identified as putative biomarkers in our study. Although the techniques used in our study were similar to those used by Yang et al., the discrepancy in these results may be due to differences in preparation of the raw saliva samples. In the Yang method, the saliva was desalted, lyophilized, and dissolved in urea sample solution, then precleaned using the PlusOne 2-D Clean-up kit; however, we did not utilize this purification method in our study [13].

Nevertheless, our study detected an elevated level of fibrinogen fragment D and complement component C3c in the saliva of OLP patients, compared with healthy control subjects. To our knowledge, this work is the first to detect changes in salivary fibrinogen D fragment and complement component C3c in OLP patients.

Fibrinogen is a soluble 340-kDa glycoprotein with three specific domains, including two outer D domains (67 kDa) and a central E domain (33 kDa) [30]. The specific function of fibrinogen in OLP and other vesiculobullous diseases is unknown. However, recent reports have described the effects of fibrinogen on vascular cells, including increased vascular tone, endothelial disorganization, and increased endothelial permeability to albumin [30]. In vitro, fibrinogen has been shown to trigger cytokine and chemokine secretion in several types of cells, and to mediate attachment of monocytes/leukocytes to the endothelium [30, 31]. In addition, the in vivo release of proinflammatory and chemotactic factors, such as monocyte chemoattractant protein-1 and interleukin 6, has been linked to binding of fibrinogen to the vascular wall [32]. Although fibrinogen is primarily synthesized by hepatocytes, the oral epithelium may synthesize and secrete fibrinogen locally in response to proinflammatory mediators, in a manner similar to that of the lung alveolar epithelium [33, 34].

The complement system is a key part of innate immune system, which aids antibodies and phagocytic cells in eradicating pathogens from an organism. Complement component C3 is a 185-kDa glycoprotein that belongs to the α2-macroglobulin family; it is the most abundant complement protein in serum [35]. Although most complement factors exhibit short half-lives, complement C3c is the major stable conversion product of complement C3, and has therefore been used as a surrogate for complement C3 in studies of complement function [36].

Fibrinogen expression at the basement membrane, and the specific pattern of C3 deposition, are both hallmarks in the diagnosis of OLP using DIF [5]. Our findings are consistent with these prior studies, suggesting that both fibrinogen and C3c may be useful salivary markers for OLP. Notably, these findings must be confirmed in a larger group of OLP patients.

Our study is the first to demonstrate decreased expression of salivary cystatin SA in OLP patients. In a previous study, Hu et al. [37] reported a reduction in the expression of salivary cystatin SA in patients with primary Sjögren’s syndrome, compared with healthy control subjects. Moreover, others have reported reduced expression of salivary cystatin SA in the saliva of periodontitis patients, compared with healthy control subjects [38, 39]. Interestingly, Ibrahim et al. [40] recently reported an increase in the expression of cathepsin L in skin specimens from patients with cutaneous LP, compared with controls. Further, this study suggested that cathepsin L could be an essential protease that contributes to the pathogenesis of LP [40]. In another study, Baron et al. showed that salivary cystatin SA can inhibit human cathepsin L and may be involved in proteolytic events in vivo [41]. Therefore, it is possible that proteolytic events may contribute to the pathogenesis of OLP, and that reduced inhibition of these proteolytic events may contribute to the destruction of basal cells in OLP. However, additional studies are required to confirm this hypothesis.

Although we investigated proteins that appear most likely to play a role in the pathogenesis of OLP, some questions still remain. According to a systematic review of MS-based proteomic studies that endeavored to define salivary biomarkers related to specific diseases, some listed biomarkers were not specific for the diseases studied—variations existed in the expression of the same protein in the same disease across multiple studies [7]. This suggests that, instead of using a single protein for diagnosis and monitoring of a specific disease, a combination of biomarkers should be utilized [7]. Based on an assessment of all salivary biomarkers, there are three categories: (1) biomarkers that are specific for a disease, (2) biomarkers that are not specific for a disease, but may indicate an abnormal condition, and (3) biomarkers that do not reliably indicate abnormality but represent diversities or variations between control and diseased samples, sample treatment protocols, or mass spectrometry platforms [7]. Our results suggest that although these three proteins (complement C3c, fibrinogen fragment D and cystatin SA) are differentially expressed in the saliva of OLP patients, they may not be specific for OLP; however, the expression of this group of proteins may be used as a multifactorial biomarker for the diagnosis of OLP and subsequent monitoring of the progression of disease. We are currently gathering more OLP patients and evaluating the sensitivity and specificity of these three biomarkers for the diagnosis of OLP. Further studies of the expression of these proteins in OLP patients, both before and after treatment, are ongoing; this may yield some insight on the ability of these proteins to serve as biomarkers of OLP.

There are some limitations to our study. First, the method that we used, 2D-PAGE followed by LC-MS/MS, might not provide the sensitivity of other recent techniques, such as MALDI-TOF or surface-enhanced laser desorption/ionization (SELDI)-TOF, both followed by LC/MS/MS [7, 42]. It has been shown that platforms based on 2D electrophoresis are affected by poor reproducibility; to avoid bias, it is often necessary to run multiple replicates of the same sample [42]. In our study, we first performed 2D-PAGE in five pairs of saliva samples from patients and controls; we found non-redundant protein spots that were differentially expressed in each pair of gels. Hence, we decided to pool saliva samples and again perform 2D-PAGE in triplicate then we chose the best pair of gels for LC-MS/MS. Moreover, it has been suggested that the best method for robust biomarker identification is analysis of multiple samples using different proteomic methodologies [7, 43]. We are currently performing shotgun proteomic analyses on saliva samples of OLP patients and comparing these with saliva from healthy control participants; we expect confirmation of whether these proteins can reliably serve as potential markers for OLP.

## Conclusion

Although our sample size of OLP patients is small, we show novel findings that the combination of these three salivary proteins (complement component C3c, fibrinogen fragment D, and cystatin SA) may serve as salivary biomarkers for diagnosis of OLP. Further studies, with a larger group of OLP patients and a before/after cohort study, are underway.

## Abbreviations

2D-PAGE: Two-dimensional polyacrylamide gel electrophoresis
DIF: Direct immunofluorescent
ELISA: Enzyme-linked immunosorbent assay
HNP-1: Defensin-1
IEF: Isoelectric focusing
IgA: Immunoglobulin A
IgJ: Immunoglobulin J
LC: Liquid chromatography
LC-MS/MS: Liquid chromatography coupled with tandem mass spectrometry
LP: Lichen planus
MALDI-TOF/TOF: Matrix assisted laser desorption/ionization time-of-flight/time-of-flight
MS: Mass spectrometry
MS/MS: Tandem mass spectrometry
NCBI: National Center for Biotechnology Information
NGAL: Neutrophil gelatinase-associated lipocalin
OLP: Oral lichen planus
PLUNC: Palate, lung, and nasal epithelium carcinoma-associated protein
REU: Reticular-Erythematous-Ulcerative
SDS-PAGE: Sodium dodecyl sulfate polyacrylamide gel
SELDI: Surface-enhanced laser desorption/ionization
sIgA: Secretory-IgA
WUS: Whole unstimulated saliva
